# Docosahexaenoic Acid, Inflammation, and Bacterial Dysbiosis in Relation to Periodontal Disease, Inflammatory Bowel Disease, and the Metabolic Syndrome

**DOI:** 10.3390/nu5083299

**Published:** 2013-08-19

**Authors:** Maria Tabbaa, Mladen Golubic, Michael F. Roizen, Adam M. Bernstein

**Affiliations:** Cleveland Clinic, Wellness Institute, 1950 Richmond Road/TR2-203, Lyndhurst, OH 44124, USA; E-Mails: tabbam@ccf.org (M.T.); golubim@ccf.org (M.G.); roizenm@ccf.org (M.F.R.)

**Keywords:** inflammation, periodontal disease, inflammatory bowel disease, metabolic syndrome, bacterial dysbiosis, microbiome, DHA, docosahexaenoic acid, omega-3, fattyacid

## Abstract

Docosahexaenoic acid (DHA), a long-chain omega-3 polyunsaturated fatty acid, has been used to treat a range of different conditions, including periodontal disease (PD) and inflammatory bowel disease (IBD). That DHA helps with these oral and gastrointestinal diseases in which inflammation and bacterial dysbiosis play key roles, raises the question of whether DHA may assist in the prevention or treatment of other inflammatory conditions, such as the metabolic syndrome, which have also been linked with inflammation and alterations in normal host microbial populations. Here we review established and investigated associations between DHA, PD, and IBD. We conclude that by beneficially altering cytokine production and macrophage recruitment, the composition of intestinal microbiota and intestinal integrity, lipopolysaccharide- and adipose-induced inflammation, and insulin signaling, DHA may be a key tool in the prevention of metabolic syndrome.

## 1. Introduction

The anti-inflammatory properties of omega-3 (omega-3) and omega-6 (omega-6) polyunsaturated fatty acids (PUFAs) have been extensively studied for their role in preventing and treating conditions such as coronary artery disease, diabetes, inflammatory bowel disease, Alzheimer’s disease, bipolar disorder, schizophrenia, and cystic fibrosis [1,2,3,4,5,6,7,8,9]. It has long been recognized that omega-3 PUFAs help decrease the production of arachidonic acid (AA)-derived pro-inflammatory prostaglandins and leukotrienes [10]. Only recently, however, has it been discovered that *resolvins* and *protectins*, two types of lipid mediators derived from docosahexaenoic acid (DHA), a omega-3 PUFA, modulate the inflammatory response, and not merely by decreasing cytokine production and dampening inflammation [11], but by actively promoting the resolution of inflammation [12]. This discovery has helped particularly with the understanding by which DHA supplementation ameliorates the inflammatory condition of periodontal disease.

That DHA supplementation helps with oral and gastrointestinal diseases in which inflammation and bacterial dysbiosis (imbalance in the natural flora) play key roles raises the question of whether DHA may assist in the prevention or treatment of other inflammatory conditions, such as the metabolic syndrome [13]. The metabolic syndrome is a complex of conditions—including dysglycemia, raised blood pressure, elevated triglyceride levels, low high-density lipoprotein cholesterol levels, and obesity (particularly central adiposity)—which raises the risk for cardiovascular disease and diabetes [14]. To date, however, this area of study has received little attention.

Here we review established and investigated associations between DHA, periodontal disease, and inflammatory bowel disease, explore the emerging science linking obesity and metabolic syndrome with the microbiome, and discuss ways in which DHA supplementation may play a key role in their management. To do so, we searched PubMed through April, 2013 for peer-reviewed systematic reviews, meta-analyses, clinical trials, experimental studies using key words such as *docosahexaenoic acid*, *bacteria*, and *bacterial infections*. We focus specifically on the role that DHA may have on the composition of gut microbiota, lipid metabolism, intestinal integrity, and chronic inflammation, and arrive at conclusions with clinical relevance for the management of metabolic syndrome.

## 2. DHA, Periodontal Disease, and Inflammatory Bowel Disease

Periodontal disease (PD) is characterized by chronic inflammation of gingival tissues resulting from dental-plaque-induced infection [15,16]. The characteristic tissue destruction results not from the pathogenic microorganisms, but from the host immune response and, therefore, the aim of therapy is to attenuate neutrophil-mediated tissue injury and monocyte infiltration and restore periodontal tissue health [11,17,18]. Nutritional supplementation, including omega-3 PUFAs, has been suggested as a therapy for the condition [19] and 900 mg of DHA and eicosapentanoic acid (EPA) supplementation (approximately 50% of each; not reported in manuscript but noted in personal communication with senior author) in combination with low-dose aspirin has been shown to be an effective therapy, as evidenced by gains in the number of gingival pockets with pocket depth <4 mm (79.5% in the omega-3 treatment group *versus* 54.7% in control) following six months of treatment [11]. In addition, approximately 25% fewer gingival sites were observed to require further oral treatment among patients taking omega-3 PUFA supplementation with aspirin compared to those who were not taking supplements [11]. One study also indicated that omega-3 supplementation with aspirin may be effective at modulating the cytokine profile of interleukin-1β (IL-1β) and interleukin-10 (IL-10) in gingival crevicular fluid (the serum transudate found in the gingival sulcus) [17].

The anti-inflammatory benefits of omega-3 PUFAs in PD may also be attributed to the products of aspirin-triggered DHA metabolism. Aspirin enhances the endogenous lipoxygenase-catalyzed hydroxylation of arachidonic acid, which in turn produces *resolvins* and *protectin D1* through acetylation of the cyclooxygenase-2 enzyme [20]. *Protectin D1* has been shown to reduce neutrophil recruitment in periodontitis in a dose-dependent fashion [17].

Modulation of these inflammatory pathways may similarly explain how DHA helps treat Inflammatory Bowel Disease (IBD), including Crohn’s Disease and Ulcerative Colitis. With IBD, the impaired ability of Pattern Recognition Receptors (PRRs), such as Toll-like receptors (TLRs), on epithelial and immune cells in the intestine to differentiate between pathogenic and commensal bacteria leads to prolonged activation of nuclear factor-κB, a pro-inflammatory transcription factor which triggers overproduction of inflammatory cytokines, such as tumor necrosis factor (TNF) and IL-1β [21]. Inflammation of the gastrointestinal tract in turn interrupts the natural balance between the mucosal immune system and normal gut microbiota [22]. Symptoms resulting from chronic inflammation include pain, bleeding, and altered bowel habits, and there is an increased risk for bowel cancer [23].

The primary goal of IBD treatment is to improve patient symptoms, induce and maintain a steroid-free clinical remission, improve quality of life, and minimize toxicity from treatment [24,25]. The omega-3 fatty acids, α-linolenic acid (ALA), EPA, and DHA may be a part of treatment, as all have been shown to inhibit the nuclear factor-κB pathway in experimental models [10]. However, a systematic review of six trials exploring the efficacy of omega-3 supplementation for maintenance of remission in Crohn’s disease was unable to confirm a therapeutic effect [4]. Although a statistically significant benefit of omega-3 PUFAs in reducing relapse in Crohn’s disease was observed (RR 0.77; 95% confidence interval (CI): 0.61–0.98; *p* = 0.03), the systematic review concluded that studies were clinically and statistically heterogenous, there was possible publication bias, and that omega-3 fatty acids, while safe, are probably ineffective for maintenance of remission in Crohn’s disease [4]. Similarly, a pooled analysis of three studies using omega-3 supplementation (with a combination dose of EPA and DHA) of at least 5 g/day for Ulcerative Colitis showed no significant difference between treatment and control groups (RR 1.02; 95% CI: 0.51–2.03; *p* = 0.96) [3].

Such inconclusive findings on the relationship between omega-3 PUFAs and IBD may be due to methodological or study population differences between studies [10]. For instance, since conversion of both the omega-3 α-linolenic acid and the omega-6 linoleic acid to long chain PUFAs requires desaturation by the same rate-limiting delta-6 desaturase enzyme, omega-3 supplement metabolism is greatly influenced by baseline, or usual, dietary fat intake of study participants [26]. Differences in baseline diets may thus lead to different outcomes among studies. Genetic differences in PUFA receptors may also affect responsiveness to fatty acid supplementation [10], and individual alterations in G-protein receptors [21] and PPAR-α, a nuclear receptor considered to be a dietary lipid sensor, have both been shown to affect the efficacy of omega-3 nutritional interventions [10]. Moreover, the ethyl ester (EE) version of omega-3 fatty acids have different effects than the native TG form found in fish and some supplements and thus different formulations may lead to divergent results [27].

## 3. DHA and the Metabolic Syndrome

Increasing the ratio of omega-3 to omega-6 PUFAs is considered part of desired dietary modifications for patients with coronary heart disease (CHD) and a combination of EPA and DHA of 0.5 g/day is used for CHD prevention, with 1.0 g/day used for CHD treatment [9]. Evidence of the efficacy of omega-3 PUFAs for hyperlipidemia, a risk factor of CHD and a component of the metabolic syndrome (Met-S), is supported by a recent systematic review of 17 trials [28]: omega-3 PUFAs, at doses of 1 g or more for at least three months, produced significant reductions in serum triglycerides of 7%–25% and higher doses (e.g., 3 grams or more per day) could produce further reductions but also raise other risk factors, such as LDL-cholesterol [28]. Fish oil may reduce blood pressure and perhaps do so in a dose-response manner (−0.66/0.35 mmHg per gram of omega-3 fatty acid); however, the antihypertensive effect of doses less than 0.5 g/day remain uncertain [29,30]. One recent study was unable to detect a beneficial effect of fish oil supplementation on inflammatory markers of Met-S, including monocyte chemotactic protein-1 (MCP-1), interleukin-6 (IL-6), and soluble intercellular adhesion molecule (sICAM-1) [31] and no change in insulin sensitivity or glucose metabolism was observed in overweight and obese adults supplemented with 2g/day of algal DHA for 4.5 months [32]. Thus, DHA may have a limited effect on the metabolic syndrome once established. However, it may play a role in its prevention.

Changes to gut bacteria have been suspected to play a role in obesity, fatty liver, diabetes, and cardiovascular disease [33,34,35,36,37,38]. They may also be a factor in the onset and progression of the metabolic syndrome through changes in energy metabolism, metabolite production of short chain fatty acids (SCFAs), intestinal wall integrity, and interactions with host immune cells [39,40]. For example, certain bacterial species ferment undigested carbohydrate or fiber to SCFAs, such as butyrate, which in turn bind to free fatty acid receptors 2 and 3 (FFAR2 and FFAR3), which are involved in the regulation of the appetite-control hormones, peptide YY and glucagon-like peptide 1 [40]. Moreover, both obesity and the metabolic syndrome have been found to be associated with increased levels of circulating lipopolysaccharide (LPS) [41]. LPS, an endotoxin released from the cell wall of gram-negative bacteria, is absorbed from the gut and enters the circulatory system, likely via chylomicron-facilitated transport or extracellular leakage through tight junctions in the epithelium [42]. When bound to PRRs such as toll-like receptor 4 (TLR4) on immune cells, LPS induces inflammation through production and release of cytokines, leukotrienes, and prostaglandins [43]. Chronic low-grade inflammation, characterized by elevated levels of C-reactive protein (CRP), contributes to the development of Met-S, and the secretion of pro-inflammatory cytokines (such as IL-6 and TNF-α) and free fatty acids from adipose tissue then heighten the inflammation [44]. Excessive adipose tissue expansion, as with obesity, heightens immune response [44] and is associated with infiltration of activated macrophages into adipose tissue [45]. Since TLRs are embedded on adipose cells, circulating LPS may directly interfere with insulin sensing [43].

The epithelial integrity of the gut is thus critical in preventing this cascade of events. Fish oil, a major source of DHA, appears effective in enhancing intestinal integrity in animal models [46], and thus may be beneficial in preventing or treating Met-S. For example, following intravenous *Escherichia coli* LPS injections into animal models, DHA supplementation was found to significantly enhance protein expression of intestinal tight junction proteins, including *occludin* and *claudin-1* [46]. DHA was also seen to improve intestinal tissue morphology, including greater villus height, decreased plasma diamine oxidase activity (DOA), and increased mucosal DOA activity in following LPS-induced inflammation [46].

DHA supplementation has also been shown to decrease LPS-induced systemic inflammation [47,48]. Following *Escherichia coli* LPS injection into animal models, DHA supplementation down-regulated mRNA expression of intestinal TLR4 [46]. Lipid-derived mediators also modulated inflammation by differentially altering the response of human macrophages to LPS [49]: for example, 17(*R*)-*Resolvin D_1_* helped resolve LPS-driven inflammation by reducing the macrophage pro-inflammatory response to LPS associated with *E. coli* and was associated with greater TNF production to assist in clearing the pathogenic bacteria [49]. Animal models have also shown that DHA-derived lipid mediators may be effective in alleviating inflammation induced by excessive adipose tissue while also improving insulin resistance [12]. Administration of *Resolvin D_1_*, formed from DHA through 15-lipoxygenase action or acetylated COX-2 [12], restores insulin sensitivity [45] and both *Resolvin D1* and *Protectin D1* induce adiponectin expression in animal models [45]. Low levels of adiponectin are associated with obesity and type 2 diabetes [50].

**Figure 1:**
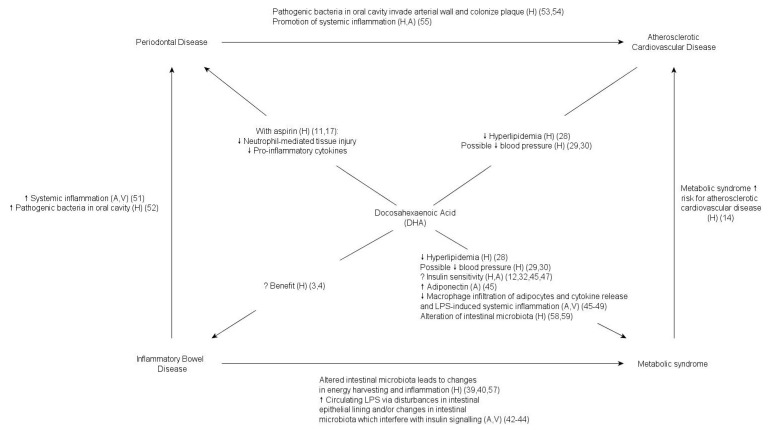
Established and proposed relationships between docosahexaenoic acid, periodontal disease, inflammatory bowel disease, metabolic syndrome, and atherosclerotic cardiovascular disease. A = Animal study; V = *In vitro* study; H = Human study.

Thus, DHA may help in prevention of the metabolic syndrome by improving intestinal integrity, improving insulin sensitivity, and decreasing LPS- or adipose-associated systemic inflammation (Figure 1).

## 4. Pathogenic Bacteria, the Microbiome, and DHA

DHA may also help with prevention of Met-S by altering intestinal microbiota, and understanding the relation between PD, IBD, and the microbiota may help illuminate potential pathways [51]. Patients with IBD have been shown to have significantly less oral microbiota diversity and a predominance of bacterial species related to opportunistic infections than patients without IBD [52]. In addition, PD patients with IBD possess higher percentage of pathogenic bacteria in oral sites of infection than PD patients without IBD [52]. Together, these data suggest intestinal microbiota impact the oral microbiome. In addition, and importantly, certain oral bacteria, such as *Porphymonas gingivalis*, a bacterial species commonly reported in PD, have been found in atherosclerotic plaques [53,54], suggesting that such bacteria travel systemically and may be involved with cardiometabolic disease development [55].

An altered balance of normal gut microbiota populations are observed in obese patients, with a higher percentage of *Firmicutes*, as opposed to *Bacteroidetes*, when compared to normal weight individuals [40,56]. Diet-induced obesity in animal models correlates with declines in the number of *Bifidobacterium* species [56]. Reduced *Bifidobacteria* have also been found to correlate with increases in inflammatory markers and insulin resistance [39]. However, it is possible that the observed microbial changes occur in a bi-directional manner, with obesity and insulin resistance being a part of the initiation and consequence of these conditions [57].

DHA can alter host microbial populations, as evidenced from infant and pediatric studies [58,59]. Breast milk is rich in omega-3 fatty acids and *Bifidobacteria* is more abundant in breastfed infants compared to formula-fed infants, suggesting that omega-3 PUFAs may influence early gut colonization [58,59]. One prospective study found fish oil supplementation delayed the age-associated increase in dominant *Bacteriodetes* among children 9–18 months old, with more pronounced changes seen in children who had ceased breast-feeding prior to the start of the intervention [58]. In this study, the authors concluded that the microbiome is still developing in 9–18 months of age and that fish oil supplementation can influence changes in the abundance of major bacterial groups among children who had stopped breast-feeding [58].

Interestingly, in a reciprocal fashion, gastrointestinal bacterial composition also affects host fatty acid metabolism [60,61]. Animal models show that administration of *Bifidobacterium* species in combination with ALA results in higher concentrations of EPA in liver tissue and DHA in brain tissue than that achieved without bacterial administration [60,61]. Thus, adequate tissue DHA may not be able to be achieved with supplementation alone if bacterial populations to metabolize the fatty acid are not in place.

Notably, there appear few reports on adverse effects of DHA consumption. Some fish oil supplements, which may be sources of DHA, have been found to be contaminated with persistent organic pollutants [62,63]. Any DHA supplementation should be done under a physician’s supervision to monitor for side effects, adverse reactions, and potential interactions with other medications.

## 5. Conclusions

Understanding the role of DHA supplementation in treating conditions such as periodontal disease and inflammatory bowel disease contributes to an appreciation of ways in which DHA may impact other diseases characterized by inflammation and bacterial dysbiosis. By beneficially altering cytokine production and macrophage recruitment, the composition of intestinal microbiota and intestinal integrity, LPS- and adipose-induced systemic inflammation, and insulin signaling, DHA may be a useful tool in the prevention of metabolic syndrome. Future research with well-conducted clinical trials is needed in this important area, as well as on the interconnectedness between oral and intestinal pathogens and host bacteria and on the bi-directional relationship between DHA and microbiota. Such research may show that DHA, prominently found in fish and algal oil, and converted from dietary sources of ALA, may have a role in curbing the global obesity and diabetes epidemics.
